# We are the Sensors of Consciousness! A Review and Analysis on How Awakenings During Sleep Influence Dream Recall

**DOI:** 10.2147/NSS.S506461

**Published:** 2025-04-30

**Authors:** Benjamin Stucky

**Affiliations:** 1 Institute of Pharmacology and Toxicology, University of Zurich, Zurich, Switzerland

**Keywords:** dreaming, serial awakenings, experience, awareness, memory, attention, questionnaire, phenomenology

## Abstract

**Purpose:**

Since the 1930s, researchers have awakened people from different stages of sleep to record what they have experienced. While some aspects, including asking whether participants had dreams or thoughts before awakening, largely remain the same, others, such as the method of awakening, vary greatly. In addition, it is often assumed that the influence of participant characteristics, such as personality traits, motivation, memory, and attention, is reduced by collecting experiences immediately after they occur, rather than through delayed morning recall. However, the extent to which these variables influence dream recall upon awakening has not yet been thoroughly investigated.

**Materials and Methods:**

To explore possible contextual and individual influences, this review analyzed 69 awakening studies conducted between 2000 and 2024 and utilized the DREAM database. Differences between sleep stages were quantified and experiences analyzed using the categories “with recall”, “without recall”, and “no report”.

**Results:**

Similar levels of null reports were found in NREM stage 2 and stage 3. Significant factors affecting dream recall included the method of awakening (lower recall with an alarm compared to calling the participant’s name), the number of study days (reduced recall for multiple days) and the sleep environment (higher recall at home compared to the laboratory), along with participant characteristics beyond age, sex and study design. Recall rates from NREM sleep are particularly sensitive to the method of awakening and interindividual differences.

**Conclusion:**

Both the awakening procedure and participant characteristics influence the amount of reported sleep experiences, which can impact study outcomes, such as the identification of neural correlates of consciousness. Therefore, greater emphasis needs to be placed on how experiences are collected and on participant characteristics, such as openness to experience or familiarity with different states of consciousness.

## Plain Language Summary

Have you ever wondered how often you dream while you sleep? We reviewed 69 studies where people were woken up to share their experiences. They reported dreaming most of the time, even in deep sleep. You might ask, “Why don’t I remember much in the morning then?” Well, maybe you forgot your dream because a lot of time has passed. These studies capture the experience right after it happens, but even then, some people remember dreams more than others. This could be because they are more focused on their dreams. How you wake up also matters. If someone shakes you awake, you may forget your dream, but if they gently call your name, you have a better chance of remembering it. You might also fail to recognize more subtle experiences, such as a sense of calm. So perhaps sleep is not as unconscious as we thought, leaving much to be explored in future studies.

## Introduction

### Early Dream Research with Awakenings

In 1937, not long after the first human electroencephalogram (EEG) recording,^1^ Loomis, Harvey and Hobart published their pioneering work on classifying sleep stages.^2^ In that same paper, they collected an experience report after a doorknob twist woke up a study participant. They assumed that the sound was incorporated into the participants’ dream, since the experiential report read: “someone had made a noise like a train.” The doorknob twist happened when the participant was in what we now call non-REM (NREM) stage 3 (N3). Even though the authors attributed the dream to one minute of lighter sleep occurring between the sound and the collection of the experience report, it could mark the first confirmed NREM sleep experience.

Just one year later, another experiment by the same authors, Davis and Davis, investigated multiple spontaneous and sometimes provoked sleep onset experiences.^3^ There we find a description of one of the earliest ‘white dreams’ from a provoked NREM stage 2 (N2) awakening. A “white dream”, or an experience without recall, is the impression of having just experienced something but not being able to remember any details. This work along with another landmark paper by Blake, Gerard, and Kleitman laid the groundwork for studies provoking multiple awakenings within a sleep episode.^4^

### Dreaming Occurs in Both REM and Non-REM Sleep

It was not until the early 1950s that these types of laboratory-based awakenings with subjective reports were taken up again in a rigorous manner.^5^ The seminal paper on REM sleep, characterized by wake-like EEG patterns and rapid eye movements with closed eyelids, suggested that dreams mainly occur in that stage.^6^,^7^ This finding was further consolidated by a large study with multiple participants, multiple nights and multiple awakenings per night that reported dreams in 80% of the REM sleep awakenings but only 7% from NREM sleep.^8^ Another paper by Dement and Wolpert^9^ used brief awakenings to associate eye movement directions with the reported gaze in the dream. All of this reinforced the view, still common in popular culture today, that dreams occur only in REM sleep.

But this idea was met with contrary evidence early on. In a study from 1959, participants who recalled dreams on most nights, so called high recallers, had on average 53% dream recall from NREM sleep compared to low recallers with 17%.^10^ Over time, accumulating evidence showed that recalling experiences from NREM sleep is not uncommon, finding experiences in up to 70% of the awakenings.^11^ Despite that, the idea of REM sleep being dream sleep did not suddenly disappear; instead, there were attempts to explain NREM dreams as occurring during preceding REM sleep or the awakening process itself.^12^ Such arguments were weakened by Foulkes, who showed that dreams can be recalled in the absence of REM episodes.^11^ He aimed to capture a broader range of experiences by introducing the now ubiquitous question “What was going through your mind?” instead of “What were you dreaming?”. Furthermore, he argued that differences in questions and the exclusion of less visual experiences might have contributed to inconsistent recall rates. In doing so, he redirected attention towards examining differences between NREM and REM experiences.^12^,^13^ Subsequently, it has been observed that REM reports tend to be longer and more vivid than NREM ones,^14–19^ though without precise delineation.^16^

### Neural Correlates of Dreaming

As it became evident that all sleep stages contain experiences, the focus shifted towards identifying transient neurophysiological changes, either common or unique to NREM and REM sleep, that account for obtaining dream reports.^20^,^21^ In this vein, Crick and Koch rejuvenated the search for neural correlates of consciousness.^22–24^ They focused on ‘phenomenal consciousness’, the felt qualities of the present moment.^25^ This definition of consciousness differs from others, as some felt states might arise without cognitive processing,^26–28^ can go unnoticed, be forgotten, or lack bodily reactions.^29–31^ In this review, we use “consciousness”, “dreaming”, and “experience” interchangeably to mean phenomenal consciousness. The subjective reality challenges physicalism, raising the question why presumably ‘non-conscious’ physical processes lead to any felt sense, known as the hard problem.^32^ While not solving this mystery,^33^ Crick and Koch argued that correlating phenomenal consciousness with neural activity is a first step.^22^ Awakening studies providing subjective reports are well-suited for this purpose^34^ and have in part led to proposed laws of consciousness.^35–37^ High-density EEG studies point to the “posterior cortical hot zone” as a correlate.^20^,^34^,^38^,^39^ However, according to a recent review, there is no consensus on the neural correlates of dreaming.^40^ A “dream catcher” experiment involving separate data gathering and analysis teams, also failed to predict dreams from the EEG better than chance at various levels of blinding.^41^ This uncertainty is further emphasized by two studies attempting to correlate the diversity of dreams with EEG signal complexity, resulting in null or conflicting findings.^42^,^43^ This raises the question: where do these inconsistencies come from?

### Diversity in Awakening Studies and Participant Characteristics

A total of 35 awakening studies conducted between 1953 and 2000 have been reviewed by Nielsen.^12^ This research indicated a mean recall rate from REM sleep of 81.9% and 43% from NREM sleep. The recall rate for REM sleep remained relatively stable over the years, but recall in NREM increased, reaching approximately 60% in the 1990s. Additionally, dream recall from N2 was found to be similar to the recall rate of NREM stage 3 (N3).^12^ Ever since then, some main ideas behind awakening protocols have largely remained consistent. This involves brief awakenings followed immediately by experience reports. Other aspects have varied considerably and their impact on dream recall remains largely unexplored.

For example, some studies provoked multiple awakenings in early sleep termed as ‘Early-Night Serial Awakenings’.^44^ Another procedure, the “Serial Awakening Paradigm”, is characterized by frequent random awakenings during the whole night.^45^ The “Sleep Interruption Technique” induces sleep onset REM or NREM through awakenings followed by brief periods of sleep and experience sampling.^46^,^47^ Not just night sleep has been under investigation, a multiple nap protocol with spontaneous awakenings as well as single nap protocols have been developed.^48–50^ Multiple awakenings have not been confined to the laboratory setting alone. Home-based awakenings utilized portable devices with algorithms to predict sleep stages^51–55^ and portable polysomnography (PSG) at home.^56^,^57^ There were also single night and multiple night studies.^58^,^59^ Questions about participants’ mental activity and dreams are the most common, with a minority asking slightly different questions,^46^,^51^,^60^,^61^ while many include secondary study-specific questions.^43^,^45^,^51^,^58^,^62^ Further variability can be found in the methods used to wake up participants, some call the participant by name,^58^,^63–65^ use an alarm,^45^,^56^,^66^ or sounds,^41^,^47^,^62^,^67^ others directly ask the question, knock or enter the room.^52^,^68–71^ Other lines of advance include interventions prior to awakenings,^72–77^ non-healthy subpopulations^56^,^57^,^62^,^71^,^78^,^79^ or pharmacological interventions.^16^

Adding to this diversity, there might be interindividual effects. Nielsen’s review already highlighted significant variability in how individuals recall experiences during deep sleep.^12^ It has been known for a while that experiences recalled during the night just after they happened can be forgotten by the same participant in the morning, highlighting the importance of memory.^80^ A recent paper found that attentional processes might even play a larger role in dream recall.^81^ Other participant characteristics associated with spontaneous morning dream recall include age, sex, attitude towards dreams, openness to experience, and creativity.^82–86^ It has been assumed that such effects are less pronounced for studies collecting the experience near to when it happens compared to delayed morning recall.^34^,^87^ But this hypothesis remains to be tested.

Given that many objectives in dream research, such as identifying neural correlates of dreaming, depend on subjective reports—which can be influenced by awakening procedures and participant characteristics—careful investigation into the effects of context and personal characteristics with a focus on awakening studies is warranted.

Our objectives are to:

1) Identify dream recall rates from studies since 2000 involving awakenings from different sleep stages and compare these rates with findings from Nielsen’s review of pre-2000 studies.^12^
2) Assess the influence of contextual variables on dream recall in awakening studies, such as the sleep environment, the questions asked, and the awakening procedure.
3) Quantify the presence of participant characteristics beyond age and gender.

To shed light on these questions, we review and analyze studies between the years 2000–2024 with awakenings from different sleep stages and subsequent experience sampling, extracting available contextual factors. For testing the presence of interindividual effects, we use the freely accessible DREAM database (v 4),^88–111^ consisting of participant level data from some of the recent laboratory-based awakening studies.‬‬‬‬‬‬‬‬‬‬‬‬‬‬‬‬‬‬‬‬‬‬‬‬‬‬‬‬‬‬‬‬‬‬‬‬‬‬‬‬‬‬‬‬‬‬‬‬‬‬‬‬‬‬‬‬‬‬‬‬‬‬‬‬‬‬‬‬‬‬‬‬‬‬‬‬‬‬‬‬‬‬‬‬‬‬‬‬‬‬‬‬‬‬‬‬‬‬‬‬‬‬‬‬‬‬‬‬‬‬‬‬‬‬‬‬‬‬‬‬‬‬‬‬‬‬‬‬‬‬‬‬‬‬‬‬‬‬‬‬‬‬‬‬‬‬‬‬‬‬‬‬‬‬‬‬‬‬‬‬‬‬‬‬‬‬‬‬‬‬‬‬‬‬‬‬‬‬‬‬‬‬‬‬‬‬‬‬‬‬‬‬‬‬‬‬‬‬‬‬‬‬‬‬‬‬‬‬‬‬‬‬‬‬‬‬‬‬‬‬‬‬‬‬‬‬‬‬‬‬‬‬‬‬‬‬‬‬‬‬‬‬‬‬‬‬‬‬‬‬‬‬‬‬‬‬‬‬‬‬‬‬‬‬‬‬‬‬‬‬‬‬

## Materials and Methods

### Study Inclusion Criteria

We have conducted a non-systematic review of studies with awakenings from different stages of sleep to collect experiential reports. The resulting list of studies aims to capture many relevant studies, though it is not meant to be exhaustive. Inclusion criteria were:

1) Experiential reports are associated with at least one sleep stage, either N1, N2, N3, NREM or REM.
2) Percentages of experiences (recall rates) are available, or inferable, for at least one stage and one experience type (“experience with recall”, “experience without recall” or “no report”).
3) Participants are healthy.
4) No interventions are present.
5) The studies were published, or made accessible on an online repository between January 2000 and July 2024.
6) Recall rates from extensive subcategories, like many collection timepoints, were omitted to avoid disproportionately increasing the weight of individual studies.
7) Whenever studies shared the same data and reported the same recall rates, only one study was retained. Sometimes the data basis was only partially shared or the same data used but complementary information reported, in which case all were included.

The studies were mainly searched on Google Scholar (https://scholar.google.com/) and PubMed (https://pubmed.ncbi.nlm.nih.gov/) with the following keywords: *dream recall, dream reports, serial awakening(s), serial awakening paradigm, serial awakening sleep, sleep interruption technique, early-night serial awakenings, sleep interruption technique, laboratory dream, laboratory awakening dream, laboratory awakening sleep, awakening sleep, awakening dream, consciousness sleep.*

Additionally, some studies were found through the citation list of other studies and a small amount through queries into Microsoft Copilot, an AI assistant.

### Extracted Variables from Studies

Whenever available, we searched and extracted the following information within the main paper or the supplementary information of each study:

1) Percentages of experience with recall, experience without recall or no report. In cases where experience with and without recall were merged, the no report category was inferred (ie, 100% - percentage of experience with and without recall).
2) The awakening method. For balanced analyses, a factor with three levels was constructed: awakening with an alarm, buzzer, sound or tone, by calling the participant by name and a category including “other” methods.
3) The question used for experience-type categorization. The three main levels were “what went through your mind”, “what did you dream” and “other”.
4) Number of awakenings per recording with levels “repeated” or “single”.
5) The sleep environment with levels “laboratory” or “at home”.
6) The type of sleep, if it was a nap or a night sleep.
7) The study days, consisting of multiple or single experimental days.
8) Participant characteristics like mean age and percentage of females, calculated per study or study subset. If only an age range was given, the mean age was approximated by the midpoint (ie, maximum age – minimum age divided by two).
9) Number of unique participants per study or study subset.
10) Number of awakenings per study or study subset.
11) If the awakening was explicitly mentioned as being gentle.

Some values were not explicitly given, but could be inferred or approximated. Inferred values are indicated it in the supplement Table S1. Some factor levels consisted of a mixture of other levels or were uncommon, these received the label “both” or “other”.

### Data Analysis: Review

All analyses were carried out with the programming language R (v 4.3.2)^112^ in the integrated development environment RStudio (v 2024.4.2.764).^113^

Three independent linear regression models were constructed, one for “experience with recall”, another for “experience without recall” and lastly one for “no report”. In order to retain statistical power, we condensed all NREM sleep stages into a single level (consisting of NREM or N1, N2 and N3). Since only experience with recall had a substantial amount of data, we used it to construct a more extensive model with variables: stage, awakening method, question type, mean age, percent female, repeated awakenings per recording, sleep environment, study duration and sleep type. For these analyses, the “other” and “both” levels were omitted. The models for experience without recall and no report only included sleep stage as an independent variable. The p-values were corrected for multiple comparisons with the Benjamini–Hochberg method per model.^114^ Additionally, comparisons between N2 and N3 were carried out with t-tests and were also corrected with Benjamini-Hochberg. All figures report the corrected p-values, whereas tables report both, the raw and corrected p-values. Cohen’s d values and corresponding effect size categories were additionally computed from the models by the emmeans (v 1.10.2)^115^ and effect size (v 0.8.8)^116^ packages. The effect size categories were “negligible” for absolute Cohen’s d values < 0.2, “small” if 0.2 ≤ | d | < 0.5, “medium” 0.5 ≤ | d | < 0.8, and “large” if | d | ≥ 0.8.

The experience with recall model only included main effects. To check whether some interactions are important, we constructed a model with all interaction terms per sleep stage. A stepwise AIC model selection with both directions was carried out with the MASS package (v 7.3–60).^117^ The main effects were retained in each step. These analyses can be found in the supplement Table S2. Furthermore, the supplement contains analyses for potential effects of study year (Figure S1), number of awakenings per participant in single night studies (Table S3) and gentle awakenings (Figure S2) on percentages of different experience types.

### Data Analysis: DREAM Database

For the DREAM database, we only included healthy participants and sleep stages that were not labeled “Unknown” or “#N/A”. Since the “Subject.ID” variable was not fully unique between studies, we created a new unique identifier per participant and study. An additional age factor was constructed with levels less or equal than 30 years or more than 30 years of age. This cutoff point was chosen, since it corresponds to the rounded mean age of 29.5 amongst the studies.

The subset of studies containing either experience with recall, experience without recall or no report, were identified. For each such report type and sleep stage, we calculated participant-based percentages. To ensure meaningful estimates, we only kept those percentages that were based on 3 or more data points per person.

First, generalized mixed effect models with sleep stages as fixed effects and study identifier as random intercept were estimated. Since the distributions were bounded by and highly skewed towards 0% and 100%, generalized ordered beta mixed model^118^ with logit link function implemented in the glmmTMB package (v 1.1.9)^119^ were used. This model is specifically designed for bounded outcomes. The results were corrected for multiple testing with Benjamini-Hochberg and amended with Cohen’s d values and the same effect size categories as mentioned above.

Additionally, to test for participant characteristics, we made use of permutation tests. Permutation tests have the advantage of making minimal assumptions on the data.^120^ The permutation test was constructed with the standard deviation of participant-based percentages as a test statistic. This was chosen, since it is a measure of how variable participants are between themselves. Thus, one can test if the observed variability is expected due to pure chance or rather due to participant grouping. Resampling of experience labels was done 10’000 times. Importantly, the structure of sex, age category, and study ID was kept intact, meaning labels were randomly resampled only within the groupings but not between. This way one can control for these factors, and the resulting significant effects are indicative of participant characteristics beyond sex, age and other variables of a given study. After each resampling, the percentages per person were calculated again for NREM (with additionally keeping stage grouping—N1, N2, and N3—intact in permutations) and REM sleep. All permutation tests reported in the main paper have been corrected for multiple testing with Benjamini-Hochberg. To see if observed NREM effects hold true for a single sub-stage, we calculated the permutation test only on N2, which contained the largest amount of data. These additional analyses are reported in the supplement Figure S3.

## Results

### Descriptive Statistics: Review

In total, 69 studies were found who met the inclusion criteria.^16^,^18^,^19^,^34^,^42–52^,^54^,^55^,^57–62^,^64–70^,^72^,^75^,^121–157^ All studies reported experience with recall percentages and 22 studies additionally captured experiences without recall and no report, see Table 1.

**Table 1:** The Reviewed 69 Studies and Their Characteristics

Publication	Year	Method	Question	Repeated	Setting	Sleep	Days	w/ recall	w/o recall	No report
Parker et al^69^	2000	Other	Other					Yes	No	No
Takeuchi et al^47^	2001	Alarm	Mind	Repeated	Lab	Night	>1	Yes	Yes	Yes
Stickgold et al^55^	2001	Other	Mind	Repeated	Home	Night	>1	Yes	No	No
Takeuchi et al^46^	2003	Alarm	Other	Repeated	Lab	Night	>1	Yes	Yes	Yes
Palagnini et al^128^	2004	Name	Mind	Single	Lab	Nap	>1	Yes	No	No
Fosse et al^18^	2004	Other	Mind	Repeated	Home	Night	>1	Yes	No	No
Wittmann et al^65^	2004	Name	Mind	Repeated	Lab	Night	>1	Yes	Yes	Yes
St-Onge et al^123^	2005	Other	Mind	Repeated	Lab	Night	1	Yes	No	No
Strauch^134^	2005	Name	Dream	Repeated	Lab	Night	>1	Yes	No	No
Grenier et al^157^	2005	Name	Mind	Repeated	Lab	Night	1	Yes	No	No
Wamsley et al^129^	2007	Name	Mind	Repeated	Lab	Night	1	Yes	Yes	Yes
Weigand et al^67^	2007	Alarm	Mind	Both	Lab	Night	1	Yes	No	No
Daoust et al^126^	2008	Other	Mind	Repeated	Lab	Night	1	Yes	Yes	Yes
Wamsley et al^129^	2008	Name	Mind	Repeated	Lab	Nap	>1	Yes	No	No
Noreika et al^44^	2009	Alarm	Mind	Repeated	Lab	Night	>1	Yes	Yes	Yes
Lusignan et al^133^	2009	Other	Mind	Repeated	Lab	Night	>1	Yes	Yes	Yes
Lara-Carrasco et al^140^	2009	Alarm	Mind	Repeated	Lab	Night	1	Yes	No	No
Lusignan et al^70^	2010	Other	Mind	Repeated	Lab	Night	1	Yes	Yes	Yes
Wamsley et al^132^	2010	Other	Mind	Repeated	Lab	Nap	1	Yes	No	No
Wamsley et al^54^	2010	Other	Mind	Repeated	Home	Night	>1	Yes	No	No
Schredl et al^148^	2010	Name	Mind	Repeated	Lab	Night	1	Yes	No	No
Chellappa et al^50^	2011	Other	Dream	Single	Lab	Nap	>1	Yes	No	No
Kahan et al^51^	2011	Alarm	Other	Repeated	Home	Night	1	Yes	No	No
Blagrove et al^152^	2011	Alarm	Mind	Repeated	Lab	Night	>1	Yes	No	No
Marzano et al^121^	2011	Name	Dream	Repeated	Lab	Night	1	Yes	No	No
Oudiette et al^16^	2012	Name	Mind	Repeated	Lab	Night	>1	Yes	No	No
Kusse et al^141^	2012		Mind	Repeated	Lab	Nap	>1	Yes	No	No
Chellappa et al^142^	2012	Other	Dream	Single	Lab	Nap	>1	Yes	No	No
Stenstrom et al^60^	2012	Name	Other	Repeated	Lab	Night	>1	Yes	Yes	Yes
Siclari et al^45^	2013	Alarm	Mind	Repeated	Lab	Night	>1	Yes	Yes	Yes
Schredl et al^127^	2014	Other	Mind	Repeated	Lab	Night	1	Yes	No	No
Sikka et al^124^	2014	Alarm	Dream	Repeated	Lab	Night	>1	Yes	No	No
Yu^122^	2014		Mind	Repeated	Lab	Night	1	Yes	No	No
Eichenlaub et al^68^	2014	Other	Mind	Single	Lab	Night	1	Yes	Yes	Yes
Carr et al^130^	2015	Alarm	Dream	Single	Lab	Nap	1	Yes	No	No
Van Rijn et al^52^	2015	Alarm	Mind	Repeated	Both	Night	>1	Yes	No	No
Yu^136^	2016		Mind	Repeated	Lab	Night	1	Yes	Yes	Yes
Nefjodov et al^149^	2016	Other	Mind	Repeated	Lab	Night	1	Yes	No	No
Scarpelli et al^64^	2017	Name	Mind	Repeated	Lab	Nap	1	Yes	Yes	Yes
Siclari et al^34^	2017	Alarm	Mind	Repeated	Lab	Night	>1	Yes	Yes	Yes
Sikka et al^143^	2017	Alarm	Dream	Repeated	Lab	Night	>1	Yes	No	No
Solomonova et al^48^	2018	Alarm		Repeated	Lab	Nap	1	Yes	No	No
Eichenlaub et al^138^	2018	Alarm	Mind	Repeated	Lab	Night	1	Yes	No	No
Feige et al^62^	2018	Alarm	Mind	Repeated	Lab	Night	>1	Yes	No	No
Siclari et al^59^	2018	Alarm	Mind	Repeated	Lab	Night	>1	Yes	Yes	Yes
Blagrove et al^135^	2019	Alarm	Mind	Repeated	Lab	Night	1	Yes	No	No
Wamsley et al^144^	2019	Name	Mind	Repeated	Lab	Night	1	Yes	No	No
Schoch et al^145^	2019	Other	Mind	Repeated	Lab	Night	1	Yes	No	No
Zhang et al^146^	2019	Name	Mind	Repeated	Lab	Night	1	Yes	No	No
Klepel et al^150^	2019	Other	Mind	Single	Lab	Night	1	Yes	No	No
Sikka et al^155^	2019	Alarm	Dream	Repeated	Lab	Night	>1	Yes	No	No
Sterpenich et al^125^	2020	Alarm	Mind	Repeated	Lab	Night	1	Yes	Yes	Yes
Blanchette-Carrière et al^72^	2020		Dream	Single	Lab	Nap	>1	Yes	No	No
Picard-Deland et al^131^	2020	Alarm	Mind	Single	Lab	Night	1	Yes	No	No
Yu^137^	2020		Mind	Repeated	Lab	Night	>1	Yes	No	No
Vallat et al^139^	2020	Name	Dream	Single	Lab	Nap	1	Yes	No	No
Martin et al^19^	2020	Name	Mind	Repeated	Lab	Night	>1	Yes	Yes	Yes
Scarpelli et al^156^	2020	Name	Mind	Single	Lab	Night	1	Yes	No	No
Spanò et al^57^	2020	Alarm	Mind	Repeated	Home	Night	1	Yes	No	No
Stephan et al^66^	2021	Alarm	Mind	Repeated	Lab	Night	>1	Yes	Yes	Yes
Picard-Deland et al^61^	2021	Alarm	Dream	Single	Lab	Nap	1	Yes	No	No
Pires et al^151^	2021		Dream	Repeated	Lab	Night	>1	Yes	No	No
Picard-Deland et al^153^	2021	Alarm	Mind	Repeated	Lab			Yes	Yes	Yes
Aamodt et al^42^	2021	Alarm	Mind	Repeated	Lab	Nap	1	Yes	Yes	Yes
Wamsley^154^	2022	Name	Mind	Repeated	Lab	Night	1	Yes	No	No
Picard-Deland et al^58^	2023	Name	Mind	Repeated	Lab	Night	1	Yes	No	No
Juan et al^75^	2023	Alarm	Mind	Repeated	Lab	Night	1	Yes	Yes	Yes
Sebastiani et al^49^	2023	Other	Dream	Single	Lab	Nap	1	Yes	No	No
Aamodt et al^43^	2023	Alarm	Mind	Repeated	Lab	Night	1	Yes	Yes	Yes

**Notes**: The studies are listed and sorted by year of publication. In particular, the columns awakening method (method), question type (question), awakenings per recording (repeated), sleep environment (setting), type of sleep (sleep), and the study days (days) as well as the presence (yes) or absence (no) of reported percentages for experience with recall (w/ recall), experience without recall (w/o recall) or no report are presented. The awakening method “name” means calling the participant by name, the awakening method “alarm” stands for waking up with an alarm/buzzer/sound/tone, question type “mind” corresponds to “What went through your mind?”, and the question type “dream” to “What did you dream?”. In general, the label “other” indicates a category that does not fall into the main categories. Numbers in superscript in the publication column correspond to the references.

The question type “What went through your mind before the awakening?” was asked in 75% of the cases, “What did you dream?” in 19.1% and 5.9% were other questions. The awakenings were repeated within each recording in 80.9% of the studies, whereas single awakenings (17.6%) and a mixture of repeated and single awakenings (1.5%) were less frequent. The sleep environment was primarily the laboratory setting (91.2%), in comparison to at home (7.4%) and both (1.5%). Multiple and single-day studies were 44.8% and 55.2%, respectively. Most studies investigated night sleep (79.1%); others involved naps (20.9%).

The overall mean of study-based mean ages was 26.9 years with a standard deviation of 11.5 years. The overall mean percentage of females was 57% ± 21.2%. Furthermore, there were on average 27.2 ± 46.4 unique participants in a study. The overall mean of the total number of awakenings per study was 92.1 ± 135.8, amounting to roughly 3.4 ± 2.9 awakenings per person.

The reviewed studies reveal an average percentage of experiences with recall of 59.5% in NREM sleep (considering only N1, N2 or N3 yields an average of 84.9%, 53.2% and 50.8%, respectively), 83.4% in REM sleep and 95.3% in W. Experiences without recall occur on average 23.8% in NREM sleep (N1 = 10%, N2 = 22% and N3 = 28.9%), 7.1% in REM and 6.6% in W. Getting no report has values of 28% in NREM (N1 = 2%, N2 = 35.9% and N3 = 21.1%), 13.2% in REM and 3.6% in W.

### Experience Models: Review

**Figure 1:**
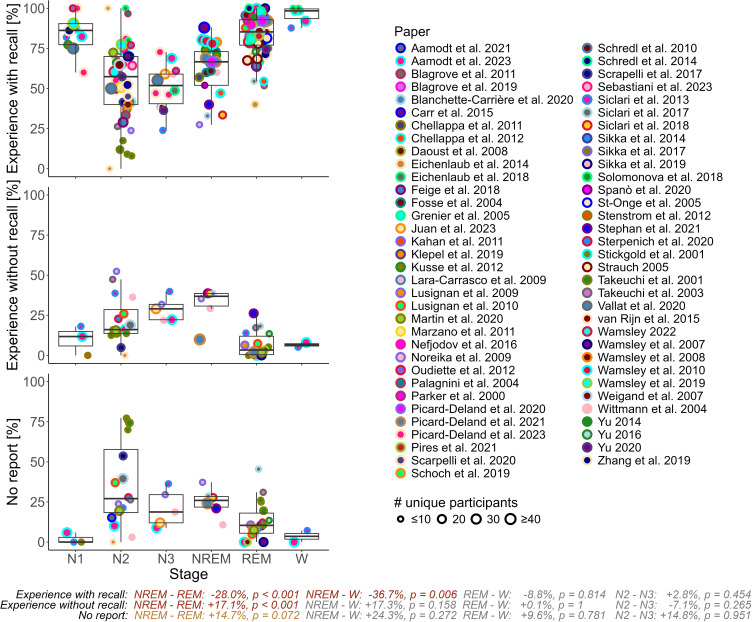
Percentages of experience type per sleep stage.All the reviewed 69 studies and their according percentages of experience with recall (top), experience without recall (middle) and no report (bottom) are shown. Some studies only use the NREM, REM and W categorization, while others further looked into N1, N2 and/or N3. The text at the bottom indicates the according linear model estimates on differences in sleep stages as well as their Benjamini-Hochberg corrected p-value. The size of the dots corresponds to the number of unique participants in a given study. The unique combination of dot fill and border color identify each study.

**Notes**: Here the individual studies’ categories NREM, N1, N2 and N3 were combined into one single NREM stage. Significant values (p ≤ 0.05) are in red, trends (0.05 < p ≤ 0.1) in orange.

**Abbreviations**: REM, Rapid eye movement; NREM, non-REM; N2, NREM stage 2; N3, NREM stage 3; W, wake.

**Figure 2:**
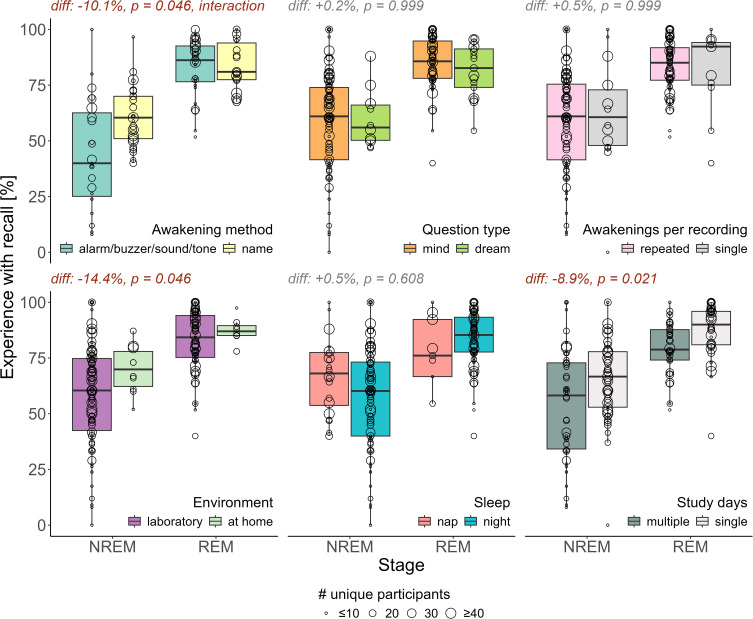
Boxplot of contextual variables.Here boxplots for the 69 reviewed studies with outcome percentage of experience with recall are shown. Furthermore, the estimated differences (diff) and Benjamini-Hochberg corrected p-values (p) from the linear model with intercept, sleep stage, awakening method, question type, mean age of participants, percentage of females, awakenings per recording, sleep environment, study days and sleep type are reported. The size of the dots corresponds to the number of unique participants in a given study.

**Notes**: Here the individual studies’ categories NREM, N1, N2 and N3 were combined into one single NREM stage. The awakening method “name” means calling the participant by name, question type “mind” corresponds to “What went through your mind?”, and the question type “dream” to “What did you dream?”. Significant values (p ≤ 0.05) are in red.

**Abbreviations**: REM, Rapid eye movement; NREM, non-REM; diff, difference; #, number of.

The experience with recall model revealed significant differences in NREM – REM (estimate = −28%, p-value corrected < 0.001, Cohen’s d = 1.43) and NREM – W (−36.7%, p = 0.006, d = −2.15), see Table 2 and Figure 1. For contextual variables the awakening method calling by name – alarm/buzzer/sound/tone (+10.1%, p = 0.046, d = 0.59), the study setting laboratory – at home (−14.4%, p = 0.046, d = −0.84) and the number of study days multiple – single (−8.9%, p = 0.021, d = −0.52) were significant, see Figure 2. The average number of awakenings per participant was not significant, see supplement Table S3. There were non-significant trends in the mean age and percentage females, but with a small effect size, see Table 2. Between N2 and N3 there were no significant differences. There was only one significant interaction between sleep stage and awakening method, where calling by name gives higher values in NREM than in REM, see Figure 2 and supplement Table S2.

**Table 2:** Models on Awakening Procedures of Reviewed Studies

Outcome	Contrast	Estimate	SE	t	p_raw_	p_cor_	s_raw_	s_cor_	d	Effect Size
w/ recall	(Intercept)	0.759	0.091	8.32	<0.001	<0.001	***	***	1.432	Large
w/ recall	NREM - REM	−0.28	0.03	−9.295	<0.001	<0.001	***	***	−1.639	Large
w/ recall	NREM - W	−0.367	0.104	−3.545	0.002	0.006	**	**	−2.152	Large
w/ recall	REM - W	−0.088	0.104	−0.841	0.678	0.814			−0.513	Medium
w/ recall	Name - alarm	0.101	0.037	2.729	0.02	0.046	*	*	0.594	Medium
w/ recall	Mind - dream	0.002	0.05	0.04	0.999	0.999			0.012	Negligible
w/ recall	Mean age	−0.003	0.001	−1.899	0.06	0.09			−0.327	Small
w/ recall	Percent female	−0.133	0.069	−1.926	0.056	0.09			−0.332	Small
w/ recall	Repeated - single	0.005	0.059	0.077	0.997	0.999			0.027	Negligible
w/ recall	Laboratory - at home	−0.144	0.054	−2.674	0.023	0.046	*	*	−0.843	Large
w/ recall	Multiple - single	−0.089	0.033	−2.744	0.007	0.021	**	*	−0.524	Medium
w/ recall	Nap - night	0.05	0.067	0.748	0.456	0.608			0.292	Small
w/ recall	N2 - N3	2.8	0.053	0.521	0.303	0.454			0.16	Negligible
w/o recall	(Intercept)	0.238	0.021	11.147	<0.001	<0.001	***	***	3.252	Large
w/o recall	NREM - REM	0.171	0.035	4.903	<0.001	<0.001	***	***	1.462	Large
w/o recall	NREM - W	0.173	0.086	2.019	0.119	0.158			1.475	Large
w/o recall	REM - W	0.001	0.087	0.017	1	1			0.013	Negligible
w/o recall	N2 - N3	−7.1	0.05	−1.423	0.088	0.265			−0.758	Medium
No report	(Intercept)	0.279	0.035	7.948	<0.001	<0.001	***	***	2.319	Large
No report	NREM - REM	0.147	0.057	2.561	0.036	0.072	*		0.764	Medium
No report	NREM - W	0.243	0.14	1.732	0.204	0.272			1.265	Large
No report	REM - W	0.096	0.143	0.672	0.781	0.781			0.501	Medium
No report	N2 - N3	14.8	0.084	1.761	0.951	0.951			0.876	Large

**Notes**: Here linear models for the 69 reviewed studies with outcomes percentage of experience with recall (w/ recall), experience without recall (w/o recall) and no report are shown. The model with recall is the most extensive with intercept, sleep stage, awakening method, question type, mean age of participants, percentage of females, awakenings per recording, sleep environment, study days and sleep type. Whereas the model without recall and no report only include sleep stage. The N2 to N3 comparison employs a complementary *t*-test on sleep depth. For each test the model estimate and its according standard error (SE), t-value (t) and p-value uncorrected (p_raw_) as well as corresponding significance stars (s_raw,_ “***” ↔ p < 0.001, “**” ↔ 0.001 ≤ p < 0.01, “*” ↔ 0.01 ≤ p < 0.05, “.” ↔ 0.05 ≤ p < 0.1, “ ‘↔ p ≥ 0.1) and Cohen’s d values (d) with their effect size category (“negligible” ↔ |d| < 0.2, “small” ↔ 0.2 ≤ |d| < 0.5, “medium” ↔ 0.5 ≤ |d| < 0.8, “large” ↔ |d| ≥ 0.8) are reported. Multiple testing corrections based on Benjamini-Hochberg are provided as corrected p-values (p_cor_) with their significance stars (s_cor_, for uncorrected values s_raw_). The awakening method “name” means calling the participant by name, the awakening method “alarm” stands for waking up with an alarm/buzzer/sound/tone, question type “mind” corresponds to “What went through your mind?”, and the question type “dream” to “What did you dream?”. “Repeated” and “single” refer to number of awakenings per recording, “laboratory” and “at home” to sleep environment, “multiple” and “single” to study days, “nap” and “night” to sleep type. Here the categories NREM, N1, N2 and N3 were combined into one single NREM stage.

**Abbreviations**: Rapid eye movement (REM), non-REM (NREM), NREM stage 2 (N2), NREM stage 3 (N3), wake (W).

For the model on experience without recall, there was a significant difference between NREM – REM (+17.1%, p < 0.001, d = 1.46). Other stage differences were not significant, even though NREM – W displays a similar Cohen’s d value of 1.48, see Table 2. No significant difference between N2 and N3 was found.

The no report model displayed only a trend between NREM – REM with a medium effect size, see Table 2. No significant difference between N2 and N3 was found.

Additional supplemental analyses on the reviewed studies revealed no significant effect of year of publication or number of participants for all experience types, but studies describing the awakening as gentle had significantly higher experience with recall percentages (+7%, p = 0.033), see supplement Figure S1 and S2.

### Participant-Level: DREAM Database

**Figure 3:**
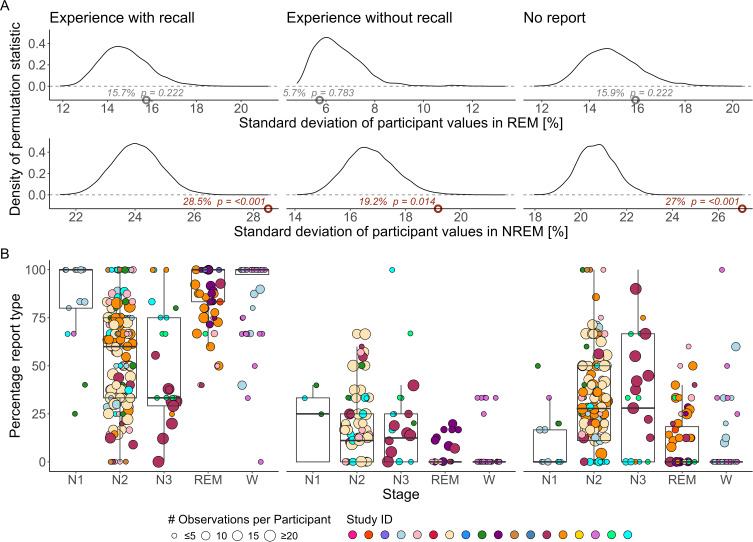
Effects of participant characteristics and percentages of experience per sleep stage in the DREAM database. (**A**) shows the density of the permutation statistic per report type (columns experience with recall, experience without recall and no report) and sleep stage (rows REM and NREM) for the DREAM database. The permutation statistic captures influences from participant characteristics that go beyond age, gender and study design as measured by the standard deviation of participant percentage values. The observed values are indicated as dots with their according value and Benjamini-Hochberg corrected p-value (p) as text. Significantly large observed values thus indicate a higher-than-chance variability in participant percentages. In (**B**) the participant-based percentages per report type (columns experience with recall, experience without recall and no report) and sleep stage (x-axis N1, N2, N3, REM and W) are displayed.

**Notes**: In (**A**) the individual studies’ categories NREM, N1, N2 and N3 were combined into one single NREM stage, permutation statistics only based on N2 can be found in the supplement. Significant values (p ≤ 0.05) are in red. The size of the dots corresponds to the number of unique awakenings per participant. The fill color of the dots indicates different studies available in the DREAM database.

**Abbreviations**: REM, Rapid eye movement; NREM, non-REM ; %, percent; #, number of.

Overall, the DREAM database included 478 participants from 18 studies. For the analysis of the DREAM database with participant-based values, the model of experience with recall displayed significant differences for N2 – REM (p < 0.001, d = −0.18), N2 – W (p = 0.003, d = −0.18), N3 – REM (p < 0.001, d = −0.22) and N3 – W (p = 0.004, d = −0.22). Other stage comparisons were not significant, nor were there any trends. The full results can be found in Table 3 and Figure 3B.

**Table 3:** Models on Sleep Stage Differences in the DREAM Database

Contrast	Estimate	SE	z	p_raw_	p_cor_	s_raw_	s_cor_	d	Effect Size
(Intercept)	−1.113	0.282	−3.953	<0.001	<0.001	***	***	−0.443	Small
N1 - N2	−0.719	0.287	−2.502	0.09	0.142			−0.111	Negligible
N1 - N3	−1.025	0.329	−3.116	0.016	0.029	*	*	−0.158	Negligible
N1 - REM	−0.04	0.311	−0.128	1	1			−0.006	Negligible
N1 - W	0.437	0.351	1.245	0.725	0.798			0.067	Negligible
N2 - N3	−0.306	0.188	−1.627	0.48	0.587			−0.047	Negligible
N2 - REM	0.679	0.156	4.355	<0.001	<0.001	***	***	0.105	Negligible
N2 - W	1.155	0.233	4.961	<0.001	<0.001	***	***	0.178	Negligible
N3 - REM	0.985	0.224	4.4	<0.001	<0.001	***	***	0.152	Negligible
N3 - W	1.462	0.279	5.233	<0.001	<0.001	***	***	0.225	Small
REM - W	0.477	0.256	1.86	0.339	0.466			0.073	Negligible

**Notes**: Here, the linear model for the DREAM database with the outcome variable percentage of experience with recall is presented. This percentage is calculated for each participant individually, provided they have three or more data points. For each test the model estimate and its according standard error (SE), z-value (z) and p-value uncorrected (p_raw_) as well as corresponding significance stars (s_raw,_ “***” ↔ p < 0.001, “*” ↔ 0.01 ≤ p < 0.05, “.” ↔ 0.05 ≤ p < 0.1, ‘↔ p ≥ 0.1) and Cohen’s d values (d) with their effect size category (“negligible” ↔ |d| < 0.2, “small” ↔ 0.2 ≤ |d| < 0.5, “medium” ↔ 0.5 ≤ |d| < 0.8, “large” ↔ |d| ≥ 0.8) are reported. Multiple testing corrections based on Benjamini-Hochberg are provided as corrected p-values (p_cor_) with their significance stars (s_cor_, with the same categorization as s_raw_).

**Abbreviations**: Rapid eye movement (REM), non-REM (NREM), NREM stage 2 (N2), NREM stage 3 (N3), wake (W).

For the DREAM database model on experience without recall, there was only a significant difference between N2 – REM (p = 0.017, d = 0.11). Furthermore, the model on no reports showed significant differences between N2 – REM (p < 0.001, d = −0.11), N2 – W (p < 0.001, d = −0.18), N3 – N1 (p = 0.029, d = −0.158), N3 – REM (p < 0.001, d = −0.15) and N3 – W (p < 0.001, d = −0.23). The results can be found in Table 3 and Figure 3B.

In NREM, 151 unique participants from 9 studies with more or equal than 3 awakenings remained. For REM, there were 70 unique participants from 7 studies with more or equal than 3 awakenings. The permutation tests to assess participant characteristics within the DREAM database were significant for NREM but not REM. Similarly, strong significances can be found for N2, see supplement Figure S3. Figure 3A displays the test statistic distributions and observed values. Experience with recall has an observed standard deviation of 28.7% (p < 0.001), experience without recall 19.3% (p = 0.014) and no report 27.1% (p < 0.001), see Figure 3A.

## Discussion

### More Slow Waves are Not Associated with Less Dreaming

A review by Nielsen on awakening studies conducted before 2000 described an increase in NREM experiences since the 1950s.^12^ Our review with studies between 2000 and 2024 revealed that awakenings from NREM sleep lead on average to 60% experiences with recall, compared to 83% in REM sleep, aligning well with Nielsen’s findings of studies in the 1990s. This indicates that the observed increase has stabilized over the past 30 years, a conclusion further supported by non-significant yearly trends in our data (see Supplement Figure S1). Furthermore, getting no report occurs rarely, but on average twice as often in N2 and N3 than in REM. The approximate 30% decrease in experience with recall in NREM compared to either REM or W is evenly distributed among increases in experiences without recall and getting no report.

At first glance, these results might suggest that N2 and N3, characterized by sleep spindles, K-complexes and slow waves, are somewhat more unconscious states than N1, REM and W. This link between slow waves and loss of consciousness has been put forth since the 1950s.^158^ One recent study found that boosting slow waves with closed loop auditory stimulation increased the experience without recall and no report conditions.

But upon closer examination of our study- and participant-level analyses, we find no significant differences between N2 and N3 sleep for each experience condition, a result in line with Nielsen’s review.^12^ Getting no report is even estimated to be slightly lower in N3 than N2. If slow waves are causally implicated in suppressing the generation of experience this result seems odd and warrants further explanation. One such reason could be that most studies focused on N2 instead of N3, which leads to reductions in statistical power. Auditory thresholds for awakening participants have been found to be highest in N3.^159^ Hence, difficulty in awakening participants from N3 sleep could result in missing null-reports and skewed percentages. However, this explanation appears inconsistent, as REM sleep has comparably high arousal thresholds—lower than N3 but higher than N2^159^—while still permitting substantial dream recall. Another potential explanation is that unmeasured study effects outweigh the influence of sleep depth. For example, studies capturing both N2 and N3 experiential reports often report slightly higher null report rates in N3 than N2.^43–45^,^65^ Nonetheless, the reported increases are small, indicating that sleep depth, quantified here as N2 and N3, is not a strong predictor for experiences during sleep. It was also suggested that local slow waves and high-frequency components occurring over posterior regions are important.^20^ But an explanation is warranted why these types of oscillations occur, as suggested by our results, with a similar frequency in N2 and N3.^75^ On the other hand a review on studies correlating delta activity with dreaming was inconclusive, where only about half of the studies report an association.^40^ Furthermore, interventional studies showing decreases in experience with recall cannot conclusively prove that experience generation is suppressed,^160^ as interventions may hinder dream encoding or retrieval.^81^,^161^ Thus, the non-significant difference between N2 and N3 could also reflect an actuality and align with observations indicating that experiences during anesthesia are not related to the depth of anesthesia.^162^ It could rather be that increases in null reports in N2 and N3 compared to N1, REM, and W in part reflect a reduced saliency of NREM experiences.^163^ Experiences in N2 and N3 might be more subtle, including experiences lacking content or self-awareness,^164^–^167^ and are thus not prominent enough to be kept in memory. Lower dream recall from N2 and N3 stages might be due to reduced arousal and attentional awareness, which are essential for successful dream recall according to the arousal–retrieval hypothesis^168^ or Conduit’s attention–based model.^169^,^170^ Additionally, the functional state-shift hypothesis suggests that memory retrieval is impaired during transitions between distinct cognitive states, as information stored in one state may not be accessible in another.^171^ In our case, sleep stages N1 and REM might resemble wakefulness more closely than N2 and N3, partially explaining our results. Further evidence for the importance of memory retrieval in dream recall comes from our results that experiences reported as forgotten dreams by participants are characteristic of N2 and N3, with a slight, non-significant increase in N3.

### Alarms, New Settings, and Demanding Study Designs Distract from Experiences

One of the main findings was that the awakening procedure is associated with dream recall rates. Generally, calling the participant by name corresponds to increases in experience with recall rates compared to using an alarm, especially when the awakenings were described as gentle. This finding is in line with the interference hypothesis,^172^ stating that dream recall can be more difficult in the presence of distractors such as an alarm clock.^173^ Greater reductions in cortisol during retrieval, likely to be observed during less stressful awakenings, were found to benefit episodic memory recall.^174^ Furthermore, it is known that the brain state from which one is awakened can persist for some time.^139^,^175^,^176^ If the awakening is gentle, the participant may remain longer in a state resembling the prior sleep experience, thereby improving recall due to the state-dependent nature of memory.^177^–^179^ This aligns with the previously discussed functional state–shift hypothesis, particularly since the effect of the awakening is only present in NREM sleep.^171^ It further underscores that higher arousal is not always beneficial for dream recall, as indicated by theories such as the activation–synthesis hypothesis,^180^ the AIM model,^13^ the arousal–retrieval model^168^ and Conduit’s attention based model.^169^,^170^ Since the awakening occurs after dream generation, the approximate 20% difference in NREM dream recall for the awakening type suggests that many null reports are not due to the absence of dream generation, but rather due to forgetting the dream. Successful long-term memory encoding has been found to require 2 minutes after the awakening,^161^ further emphasizing the need for some uninterrupted time for retrieval 34 or memorization.^173^ Future studies should thus investigate the effect of awakening protocols more thoroughly.

Furthermore, the study’s environment may influence dream recall, as suggested by the results. Home studies yielded higher recall rates, both in REM and NREM, which might be due to the participant being in a familiar sleep environment. In the laboratory, first night effects of dream recall could be present, most noticeable in studies with no adaptation nights or nonconsecutive experimental nights.^181^ Both the functional state–shift hypothesis^171^ and interference hypothesis^172^ support this finding, as the unfamiliar laboratory environment could be a distractor, causing a more abrupt shift in consciousness and thus leading to lower recall. An alternative explanation of increased dream recall in the home setting could be that home studies were often based on algorithms, which can lead to misclassifying sleep stages and in turn confound recall rates. Nonetheless, this argument is weakened by the fact that two of the studies conducted polysomnography at home and both NREM and REM sleep displayed higher recall at home.

Another effect related to the study design is that studies extending over several days display reduced dream recall. This effect might reflect poor adherence to the study protocol, likely caused by the tiring nature of undergoing multiple days of awakenings. No such effects were observed for multiple awakenings per recording. Hence, many awakenings in a single night might be less tiring than multiple days of awakenings. Since we did not quantify the exact number of awakenings for each participant per recording, excessive awakenings impairing dream recall cannot be ruled out. Again, this finding might reflect a sharper state shift^171^ or bigger distraction^172^ due to participants feeling tired or annoyed after many days of awakenings. Days of bad sleep might also alter attentional processes, supported by Conduit’s attention-based model.^169^,^170^

Another unexpected finding was that the question asked, even though having slightly lower recall for asking about dreams compared to mental activity (Figure 2), was not significant. Some of the reviewed studies may have instructed participants to report dreams, explaining that this includes any mental activity, but did not mention this clarification in the paper, thereby masking any effects. It could also be that the questions “What went through your mind?” and “What did you dream?” yield similar responses. Both wordings may imply a limited focus on thinking and imagery and miss more subtle experiences, like feeling a sense of calm.^164^,^182^,^183^ As mentioned above, states of pure awareness during sleep, lacking rich content, have been observed.^167^,^184^ However, participants might not recognize these as states of consciousness, or if they do, fail to categorize them as a dream or mental activity. Thus, refining the questions being asked remains relevant for future research.

### Participant Characteristics Affect NREM Dream Recall

Lastly, another important finding from this study suggests the presence of participant characteristics that cannot be attributed to age, gender or differences in study design. These results might be taken as evidence for people having different levels of consciousness during sleep, but they could just as well point to dream encoding or retrieval.^185^ Trait variables have been noted to potentially influence dream generation, encoding and retrieval.^185^ Numerous factors are known or thought to affect spontaneous morning dream recall, such as, regional cerebral blood flow,^68^ creativity,^83^ default mode network connectivity during sleep and wakefulness,^83^,^139^ visual memory,^186^ episodic and autobiographical memory,^187^ motivation,^188^ imagination proneness,^82^ boundary thinness,^189^ openness to experience^82^,^84^ or attitude towards dreams.^85^,^173^ All these factors are likely candidates to also influence experience sampling from awakenings. Interestingly, only NREM dream recall was significantly influenced by characteristics beyond age, gender and study design. This variation in dream recall during deep sleep has been previously observed,^190^ but it remains inadequately explained.^12^ One possibility is that participants exhibit different levels of dream generation, encoding, or retrieval specific to NREM sleep. Another hypothesis is that familiarity with various states of consciousness might make less visual states during NREM more recognizable.^191^ Future studies are needed to pinpoint the sources of these effects in NREM.

## Limitations

This review is subject to certain limitations. First, likely not all awakening studies since 2000 have been identified, potentially influencing the results. Nonetheless, numerous studies from diverse research groups have been included, ensuring a more balanced outcome. Second, only a few contextual variables were readily available for most studies, hence limiting some questions to be addressed in this review. Third, the analyses are correlational and not suited for establishing definitive causal relationships. However, these analyses paint a coherent picture and can inform strategies for optimizing future awakening studies. Last, this review and analysis, as well as current awakening studies, do not provide conclusive evidence for the degree to which the null reports reflect the absence of phenomenal experience, or whether all reported experiences are actual experiences and when exactly they happened prior to the awakening.^185^ Our results only suggest, through the observed significant contextual and participant variables, that some of the null reports are not due to being conscious or not. This needs to be further investigated and if possible quantified.

## Conclusion

Our review of 69 awakening studies and the analysis of the DREAM database indicate that contextual variables and participant characteristics play an important role in experience recall rates. In particular, the method of awakening, the sleep environment, and participant characteristics beyond age, sex and study design are crucial factors. The awakening process and interindividual differences are especially important during NREM sleep, a state of fleeting memory with perhaps subtle experiences. To find meaningful neural correlates of consciousness, one requires good-quality data on both the neural and subjective side. Hence, capturing the experiential aspect of reality is crucial and requires further investigation.

With rigorous subjective methodology in place, future research can employ machine learning to quantify the relationship between neuronal activity and experience, an area currently underexplored.^192^ Interpretable machine learning models provide insights into the aspects a model uses for decision-making^193–195^ and thus serve as investigative tools to discover neural aspects of consciousness. Given the plethora of philosophical positions on consciousness,^196^ and the potential compatibility of current neuroscientific evidence with non-physicalist theories,^197^ our search for neural correlates should remain as philosophically neutral as possible. This means in particular to avoid the assumption that there is a direct correspondence between objective and subjective measures from the outset, and instead to explore the extent of their association. To this end, one could introduce benchmarks based on the generalizability of state-of-the-art machine learning models to unseen participants.

Consciousness during sleep can also be studied more directly, for example through communicating with people in lucid dreams, ie states of consciousness where one is aware that one is dreaming while dreaming.^198^ Real-time communication between a dreamer and the external world is possible even in NREM sleep stages^199^ and might be extendable to dreamless awareness.^164^,^166^,^167^ This holds the potential for overcoming some limitations of retrospective reports. But even during direct communication with the dreamer, participant characteristics and the questions asked likely play a role in the responses one gets. Some people may be more familiar with certain states of being than others, or give skewed reports containing preconceived opinions or interpretations, rather than observing what is experienced in an impartial way.

To overcome this, one of the most promising avenues is using first-person approaches, such as the micro-phenomenological interview where individuals undergo detailed interviews that aim at enhancing precision of reports.^182^,^184^ The Indian tradition has developed rigorous subjectivity for millennia,^200^,^201^ and hence we need to engage in a cross-cultural dialogue.

Another development that led to large numbers of recalled dreams, even from N3, trained the participants in the questionnaire beforehand and clarified any uncertainties.^43^ A different study instructed participants to lie calmly in bed after the awakening with some time to let the memories come back.^34^

All of these avenues might sharpen the subjective data used in awakening studies and consciousness research in general.

In conclusion, our review of 69 studies between 2000 and 2024 and analysis of the DREAM database, in which participants were woken up to collect experience reports, shows that the context of the awakenings, such as the awakening process, the sleep environment and participant characteristics influence dream recall rates. This highlights the need for dream research to refine how reports are collected and pay closer attention to the subjective realm, where consciousness is to be found. We ourselves are the most direct sensors of consciousness, not any devices or algorithms. By refining our own “measurement instrument”, we may discover a wide range of states of consciousness in areas we previously thought none existed, enabling innovative ways of using these states and informing the treatment and care of disorders of consciousness. Or in the words of the Indian philosopher and mystic Sri Aurobindo:

“It is through consciousness, by an instrumentation of consciousness only that the nature and laws and movements of consciousness can be discovered […]”^202^

## References

[1] Tudor M, Tudor L, Tudor KI. (1873-1941)- the history of electroencephalography. *Acta Medica Croat Cas Hravatske Akad Med Znan*. 2005;59(4):307–313. 16334737

[2] Loomis AL, Harvey EN, Hobart GA. Cerebral states during sleep, as studied by human brain potentials. *J Exp Psychol*. 1937;21(2):127. doi:10.1037/h0057431

[3] Davis H, Davis PA, Loomis AL, Harvey EN, Hobart G. Human brain potentials during the onset of sleep. *J Neurophysiol*. 1938;1(1):24–38. doi:10.1152/jn.1938.1.1.24 17838964

[4] Blake H, Gerard RW, Kleitman N. Factors influencing brain potentials during sleep. *J Neurophysiol*. 1939;2(1):48–60. doi:10.1152/jn.1939.2.1.48

[5] Aserinsky E, Kleitman N. Regularly occurring periods of eye motility, and concomitant phenomena, during sleep. *Science*. 1953;118(3062):273–274. doi:10.1126/science.118.3062.273 13089671

[6] Aserinsky E, Kleitman N. Two types of ocular motility occurring in sleep. *J Appl Physiol*. 1955;8(1):1–10. doi:10.1152/jappl.1955.8.1.1 13242483

[7] Aserinsky E. The discovery of REM sleep. *J Hist Neurosci*. 1996;5(3):213–227. doi:10.1080/09647049609525671 11618742

[8] Dement W, Kleitman N. The relation of eye movements during sleep to dream activity: an objective method for the study of dreaming. *J Exp Psychol*. 1957;53(5):339–346. doi:10.1037/h0048189. 13428941

[9] Dement W, Wolpert EA. The relation of eye movements, body motility, and external stimuli to dream content. *J Exp Psychol*. 1958;55(6):543–553. doi:10.1037/h0040031 13563767

[10] Goodenough DR, Shapiro A, Holden M, Steinschriber L. A comparison of “dreamers” and “nondreamers”: eye movements, electroencephalograms, and the recall of dreams. *J Abnorm Soc Psychol*. 1959;59(3):295–302. doi:10.1037/h0040532

[11] Foulkes WD. Dream reports from different stages of sleep. *J Abnorm Soc Psychol*. 1962;65:14–25. doi:10.1037/h0040431 13894288

[12] Nielsen TA. A review of mentation in REM and NREM sleep: “covert” REM sleep as a possible reconciliation of two opposing models. *Behav Brain Sci*. 2000;23(6):851–866. doi:10.1017/s0140525x0000399x 11515145

[13] Hobson JA, Pace-Schott EF, Stickgold R. Dreaming and the brain: toward a cognitive neuroscience of conscious states. *Behav Brain Sci*. 2000;23(6):793–842. doi:10.1017/s0140525x00003976 11515143

[14] Siclari F, Bassetti C, Tononi G. Conscious experience in sleep and wakefulness. *Schweiz Arch Für Neurol Psychiatr*. 2012;163(8):273–278.

[15] Antrobus J. REM and NREM sleep reports: comparison of word frequencies by cognitive classes. *Psychophysiology*. 1983;20(5):562–568. doi:10.1111/j.1469-8986.1983.tb03015.x 6635096

[16] Oudiette D, Dealberto MJ, Uguccioni G, et al. Dreaming without REM sleep. *Conscious Cogn*. 2012;21(3):1129–1140. doi:10.1016/j.concog.2012.04.010 22647346

[17] Foulkes D, Rechtschaffen A. PRESLEEP DETERMINANTS OF DREAM CONTENT: EFFECT OF TWO FILMS. *Percept Mot Skills*. 1964;19:983–1005. doi:10.2466/pms.1964.19.3.983 14242569

[18] Fosse R, Stickgold R, Hobson JA. Thinking and hallucinating: reciprocal changes in sleep. *Psychophysiology*. 2004;41(2):298–305. doi:10.1111/j.1469-8986.2003.00146.x 15032995

[19] Martin JM, Andriano DW, Mota NB, et al. Structural differences between REM and non-REM dream reports assessed by graph analysis. *PLoS One*. 2020;15(7):e0228903. doi:10.1371/journal.pone.0228903 32701992 PMC7377375

[20] Tononi G, Boly M, Cirelli C. Consciousness and sleep. *Neuron*. 2024;112(10):1568–1594. doi:10.1016/j.neuron.2024.04.011 38697113 PMC11105109

[21] Nemeth G, Fazekas P. Beyond the REM--NREM dichotomy: a multidimensional approach to understanding dreaming. *J Conscious Stud*. 2018;25(11–12):13–33.

[22] Crick F, Koch C. Toward a neurobiological theory of consciousness. *Semin Neurosci*. 1990;2:263–275.

[23] Koch C, Massimini M, Boly M, Tononi G. Neural correlates of consciousness: progress and problems. *Nat Rev Neurosci*. 2016;17(5):307–321. doi:10.1038/nrn.2016.22 27094080

[24] LeDoux JE, Michel M, Lau H. A little history goes a long way toward understanding why we study consciousness the way we do today. *Proc Natl Acad Sci*. 2020;117(13):6976–6984. doi:10.1073/pnas.1921623117 32170012 PMC7132279

[25] Nagel T. 11 what is it like to be a bat? In: Block N editor. *Volume I Readings in Philosophy of Psychology, Volume I*. Harvard University Press; 2013:159–168. doi:10.4159/harvard.9780674594623.c15.

[26] Smith SM. Phenomenal overflow, bodily affect, and some varieties of access. *Rev Philos Psychol*. 2019;10(4):787–808. doi:10.1007/s13164-019-00436-x

[27] Block N. Perceptual consciousness overflows cognitive access. *Trends Cognit Sci*. 2011;15(12):567–575. doi:10.1016/j.tics.2011.11.001 22078929

[28] Block N. On a confusion about a function of consciousness. *Brain Behav Sci*. 1995;18(2):227–247. doi:10.1017/s0140525x00038188

[29] Demertzi A, Laureys S, Boly M. *Coma, Persistent Vegetative States, and Diminished Consciousness*. Oxford, United Kingdom: Elsevier; 2009. doi:10.1016/B978-012373873-8.00017-7

[30] Laureys S, Owen AM, Schiff ND. Brain function in coma, vegetative state, and related disorders. *Lancet Neurol*. 2004;3(9):537–546. doi:10.1016/S1474-4422(04)00852-X 15324722

[31] Goldfine AM, Schiff ND. Consciousness: its neurobiology and the major classes of impairment. *Neurol Clin*. 2011;29(4):723–737. doi:10.1016/j.ncl.2011.08.001 22032656 PMC3222861

[32] Chalmers D. The hard problem of consciousness. In: *The Blackwell Companion to Consciousness*. John Wiley & Sons, Ltd; 2017:32–42. doi:10.1002/9781119132363.ch3

[33] Schurger A, Graziano M. Consciousness explained or described? *Neurosci Conscious*. 2022;2022(1):niac001. doi:10.1093/nc/niac001 35145759 PMC8824704

[34] Siclari F, Baird B, Perogamvros L, et al. The neural correlates of dreaming. *Nat Neurosci*. 2017;20(6):872–878. doi:10.1038/nn.4545 28394322 PMC5462120

[35] Seth AK, Bayne T. Theories of consciousness. *Nat Rev Neurosci*. 2022;23(7):439–452. doi:10.1038/s41583-022-00587-4 35505255

[36] Albantakis L, Barbosa L, Findlay G, et al. Integrated information theory (IIT) 4.0: formulating the properties of phenomenal existence in physical terms. *PLOS Comput Biol*. 2023;19(10):e1011465. doi:10.1371/journal.pcbi.1011465 37847724 PMC10581496

[37] Tononi G, Boly M, Massimini M, Koch C. Integrated information theory: from consciousness to its physical substrate. *Nat Rev Neurosci*. 2016;17(7):450–461. doi:10.1038/nrn.2016.44 27225071

[38] Ihalainen R, Gosseries O, de Steen FV, et al. How hot is the hot zone? Computational modelling clarifies the role of parietal and frontoparietal connectivity during anaesthetic-induced loss of consciousness. *NeuroImage*. 2021;231:117841. doi:10.1016/j.neuroimage.2021.117841 33577934

[39] Bréchet L, Brunet D, Perogamvros L, Tononi G, Michel CM. EEG microstates of dreams. *Sci Rep*. 2020;10:17069. doi:10.1038/s41598-020-74075-z 33051536 PMC7553905

[40] Ruby PM, Yang X, Sun J, Liu P, Qin W. The neural correlates of dreaming have not been identified yet. commentary on “the neural correlates of dreaming. Nat Neurosci. 2017. *Front Neurosci*. 2020;14:14. doi:10.3389/fnins.2020.585470 33192271 PMC7662438

[41] Wong W, Noreika V, Móró L, et al. The dream catcher experiment: blinded analyses failed to detect markers of dreaming consciousness in EEG spectral power. *Neurosci Conscious*. 2020;2020(1):niaa006. doi:10.1093/nc/niaa006 32695475 PMC7362719

[42] Aamodt A, Nilsen AS, Thürer B, et al. EEG signal diversity varies with sleep stage and aspects of dream experience. *Front Psychol*. 2021;12:655884. doi:10.3389/fpsyg.2021.655884 33967919 PMC8102678

[43] Aamodt A, Sevenius Nilsen A, Markhus R, et al. EEG Lempel-Ziv complexity varies with sleep stage, but does not seem to track dream experience. *Front Hum Neurosci*. 2023;16:987714. doi:10.3389/fnhum.2022.987714 36704096 PMC9871639

[44] Noreika V, Valli K, Lahtela H, Revonsuo A. Early-night serial awakenings as a new paradigm for studies on NREM dreaming. *Int J Psychophysiol off J Int Organ Psychophysiol*. 2009;74(1):14–18. doi:10.1016/j.ijpsycho.2009.06.002 19596384

[45] Siclari F, LaRocque JJ, Postle BR, Tononi G. Assessing sleep consciousness within subjects using a serial awakening paradigm. *Front Psychol*. 2013;4:542. doi:10.3389/fpsyg.2013.00542 23970876 PMC3747360

[46] Takeuchi T, Ogilvie RD, Murphy TI, Ferrelli AV. EEG activities during elicited sleep onset REM and NREM periods reflect different mechanisms of dream generation. *Clin Neurophysiol*. 2003;114(2):210–220. doi:10.1016/S1388-2457(02)00385-1 12559227

[47] Takeuchi T, Miyasita A, Inugami M, Yamamoto Y. Intrinsic dreams are not produced without REM sleep mechanisms: evidence through elicitation of sleep onset REM periods. *J Sleep Res*. 2001;10(1):43–52. doi:10.1046/j.1365-2869.2001.00237.x 11285054

[48] Solomonova E, Dubé S, Samson-Richer A, Blanchette-Carrière C, Paquette T, Nielsen T. Dream content and procedural learning in Vipassana meditators and controls. *Dreaming*. 2018;28(2):99–121. doi:10.1037/drm0000081

[49] Sebastiani L, Barcaro U, Paradisi P, Frumento P, Faraguna U. Morning naps architecture and mentation recall complexity. *J Sleep Res*. 2023;32(5):e13915. doi:10.1111/jsr.13915 37139546

[50] Chellappa SL, Frey S, Knoblauch V, Cajochen C. Cortical activation patterns herald successful dream recall after NREM and REM sleep. *Biol Psychol*. 2011;87(2):251–256. doi:10.1016/j.biopsycho.2011.03.004 21419827

[51] Kahan TL, LaBerge SP. Dreaming and waking: similarities and differences revisited. *Conscious Cogn*. 2011;20(3):494–514. doi:10.1016/j.concog.2010.09.002 20933437

[52] van Rijn E, Eichenlaub J-B, Lewis JB, et al. The dream-lag effect: selective processing of personally significant events during rapid eye movement sleep, but not during slow wave sleep. *Neurobiol Learn Mem*. 2015;122:98–109. doi:10.1016/j.nlm.2015.01.009 25683202

[53] Ajilore O, Stickgold R, Rittenhouse CD, Hobson JA. Nightcap: laboratory and home-based evaluation of a portable sleep monitor. *Psychophysiology*. 1995;32(1):92–98. doi:10.1111/j.1469-8986.1995.tb03410.x 7878174

[54] Wamsley EJ, Perry K, Djonlagic I, Babkes Reaven L, Stickgold R. Cognitive replay of visuomotor learning at sleep onset: temporal dynamics and relationship to task performance. *Sleep*. 2010;33(1):59–68. doi:10.1093/sleep/33.1.59 20120621 PMC2802248

[55] Stickgold R, Malia A, Fosse R, Propper R, Hobson JA. Brain-mind states: I. Longitudinal field study of sleep/wake factors influencing mentation report length. *Sleep*. 2001;24(2):171–179. doi:10.1093/sleep/24.2.171 11247053

[56] Bértolo H, Paiva T, Pessoa L, Mestre T, Marques R, Santos R. Visual dream content, graphical representation and EEG alpha activity in congenitally blind subjects. *Cogn Brain Res*. 2003;15(3):277–284. doi:10.1016/S0926-6410(02)00199-4 12527101

[57] Spanò G, Pizzamiglio G, McCormick C, et al. Dreaming with hippocampal damage. *eLife*. 2020;9:e56211. doi:10.7554/eLife.56211 32508305 PMC7279885

[58] Picard-Deland C, Konkoly K, Raider R, et al. The memory sources of dreams: serial awakenings across sleep stages and time of night. *Sleep*. 2023;46(4):zsac292. doi:10.1093/sleep/zsac292 36462190 PMC10091095

[59] Siclari F, Bernardi G, Cataldi J, Tononi G. Dreaming in NREM sleep: a high-density EEG study of slow waves and spindles. *J Neurosci*. 2018;38(43):9175–9185. doi:10.1523/JNEUROSCI.0855-18.2018 30201768 PMC6199409

[60] Stenstrom P, Fox K, Solomonova E, Nielsen T. Mentation during sleep onset theta bursts in a trained participant: a role for NREM stage 1 sleep in memory processing? *Int J Dream Res*. 2012;5(1):37–46.

[61] Picard-Deland C, Aumont T, Samson-Richer A, Paquette T, Nielsen T. Whole-body procedural learning benefits from targeted memory reactivation in REM sleep and task-related dreaming. *Neurobiol Learn Mem*. 2021;183:107460. doi:10.1016/j.nlm.2021.107460 34015442

[62] Feige B, Nanovska S, Baglioni C, et al. Insomnia-perchance a dream? Results from a NREM/REM sleep awakening study in good sleepers and patients with insomnia. *Sleep*. 2018;41(5). doi:10.1093/sleep/zsy032 29432570

[63] Casey CP, Tanabe S, Farahbakhsh Z, et al. Distinct EEG signatures differentiate unconsciousness and disconnection during anaesthesia and sleep. *Br J Anaesth*. 2022;128(6):1006–1018. doi:10.1016/j.bja.2022.01.010 35148892 PMC9428919

[64] Scarpelli S, D’Atri A, Mangiaruga A, et al. Predicting dream recall: EEG activation during NREM sleep or shared mechanisms with wakefulness? *Brain Topogr*. 2017;30(5):629–638. doi:10.1007/s10548-017-0563-1 28434101

[65] Wittmann L, Palmy C, Schredl M. NREM sleep dream recall, dream report length and cortical activation. *Sleep Hypn*. 2004;6(2):54–58.

[66] Stephan AM, Lecci S, Cataldi J, Siclari F. Conscious experiences and high-density EEG patterns predicting subjective sleep depth. *Curr Biol*. 2021;31(24):5487–5500.e3. doi:10.1016/j.cub.2021.10.012 34710350

[67] Weigand D, Michael L, Schulz H. When sleep is perceived as wakefulness: an experimental study on state perception during physiological sleep. *J Sleep Res*. 2007;16(4):346–353. doi:10.1111/j.1365-2869.2007.00619.x 18036079

[68] Eichenlaub JB, Bertrand O, Morlet D, Ruby P. Brain reactivity differentiates subjects with high and low dream recall frequencies during both sleep and wakefulness. *Cereb Cortex N Y N 1991*. 2014;24(5):1206–1215. doi:10.1093/cercor/bhs388 23283685

[69] Parker JD, Bauermann TM, Smith CT. Alexithymia and impoverished dream content: evidence from rapid eye movement sleep awakenings. *Psychosom Med*. 2000;62(4):486–491. doi:10.1097/00006842-200007000-00006 10949093

[70] Lusignan FA, Godbout R, Dubuc MJ, Daoust AM, Mottard JP, Zadra A. NonREM sleep mentation in chronically-treated persons with schizophrenia. *Conscious Cogn*. 2010;19(4):977–985. doi:10.1016/j.concog.2010.09.021 20971658

[71] Daoust AM, Lusignan FA, Braun CMJ, Mottron L, Godbout R. EEG correlates of emotions in dream narratives from typical young adults and individuals with autistic spectrum disorders. *Psychophysiology*. 2008;45(2):299–308. doi:10.1111/j.1469-8986.2007.00626.x 18047484

[72] Blanchette-Carrière C, Julien SH, Picard-Deland C, et al. Attempted induction of signalled lucid dreaming by transcranial alternating current stimulation. *Conscious Cogn*. 2020;83:102957. doi:10.1016/j.concog.2020.102957 32534325

[73] Nieminen JO, Gosseries O, Massimini M, et al. Consciousness and cortical responsiveness: a within-state study during non-rapid eye movement sleep. *Sci Rep*. 2016;6:30932. doi:10.1038/srep30932 27491799 PMC4974655

[74] Darracq M, Funk CM, Polyakov D, et al. Evoked alpha power is reduced in disconnected consciousness during sleep and anesthesia. *Sci Rep*. 2018;8(1):16664. doi:10.1038/s41598-018-34957-9 30413741 PMC6226534

[75] Juan E, Arslan C, Regnath F, Talamini LM. Boosting sleep slow waves suppresses dreaming. doi:10.1101/2023.03.10.532054

[76] Cipolli C, Fagioli I, Mazzetti M, Tuozzi G. Incorporation of presleep stimuli into dream contents: evidence for a consolidation effect on declarative knowledge during REM sleep? *J Sleep Res*. 2004;13(4):317–326. doi:10.1111/j.1365-2869.2004.00420.x 15560766

[77] Schredl M, Atanasova D, Hörmann K, Maurer JT, Hummel T, Stuck BA. Information processing during sleep: the effect of olfactory stimuli on dream content and dream emotions. *J Sleep Res*. 2009;18(3):285–290. doi:10.1111/j.1365-2869.2009.00737.x 19552703

[78] Cataldi J, Stephan AM, Haba-Rubio J, Siclari F. Shared EEG correlates between non-REM parasomnia experiences and dreams. *Nat Commun*. 2024;15(1):3906. doi:10.1038/s41467-024-48337-7 38724511 PMC11082195

[79] Cipolli C, Bonanni E, Maestri M, Mazzetti M, Murri L. Dream experience during REM and NREM sleep of patients with complex partial seizures. *Brain Res Bull*. 2004;63(5):407–413. doi:10.1016/j.brainresbull.2003.12.014 15245768

[80] Cipolli C, Calasso E, Maccolini S, Pani R, Salzarulo P. Memory processes in morning recall after multiple night awakenings. *Percept Mot Skills*. 1984;59(2):435–446. doi:10.2466/pms.1984.59.2.435

[81] Blain S, de la Chapelle A, Caclin A, Bidet-Caulet A, Ruby P. Dream recall frequency is associated with attention rather than with working memory abilities. *J Sleep Res*. 2022;31(5):e13557. doi:10.1111/jsr.13557 35102655

[82] Watson D. To dream, perchance to remember: individual differences in dream recall. *Personal Individ Differ*. 2003;34(7):1271–1286. doi:10.1016/S0191-8869(02)00114-9

[83] Vallat R, Türker B, Nicolas A, Ruby P. High dream recall frequency is associated with increased creativity and default mode network connectivity. *Nat Sci Sleep*. 2022;14:265–275. doi:10.2147/NSS.S342137 35228825 PMC8881930

[84] Schredl M, Göritz AS. Dream recall frequency, attitude toward dreams, and the Big Five personality factors. *Dreaming*. 2017;27(1):49–58. doi:10.1037/drm0000046

[85] Elce V, Bergamo D, Bontempi G, et al. The individual determinants of morning dream recall. *Communications Psychology*. 2025;3. doi:10.1101/2024.05.23.595531 PMC11836467 39966517

[86] De Gennaro L, Marzano C, Cipolli C, Ferrara M. How we remember the stuff that dreams are made of: neurobiological approaches to the brain mechanisms of dream recall. *Behav Brain Res*. 2012;226(2):592–596. doi:10.1016/j.bbr.2011.10.017 22024432

[87] Cecconi B, Montupil J, Mortaheb S, et al. Study protocol: cerebral characterization of sensory gating in disconnected dreaming states during propofol anesthesia using fMRI. *Front Neurosci*. 2024:18. doi:10.3389/fnins.2024.1306344 PMC10900985 38419667

[88] Araujo D, Palhano-Fontes F, Andrade K, Mota-rolim Sergio. Brain institute - federal university of rio grande do norte. doi:10.26180/23904699.V1

[89] De Gennaro L. DeGennaro_Children&Adolescents. doi:10.6084/M9.FIGSHARE.22220701.V1

[90] Scarpelli S, De Gennaro L. DeGennaro_MULTIPLEAWAKENINGS. doi:10.6084/M9.FIGSHARE.22086266.V1

[91] Scarpelli S, De Gennaro LD. 10.6084/M9.FIGSHARE.14500563.V1

[92] Scarpelli S, De Gennaro LD. 10.6084/M9.FIGSHARE.16950856.V1

[93] Scarpelli S, De Gennaro LD. 10.6084/M9.FIGSHARE.14899506.V1

[94] Demirel C, Gott J, Dresler M. Dream Database from Donders. doi:10.6084/M9.FIGSHARE.21388722.V2

[95] Wong W. DREAM dataset package template. doi:10.26180/13301504.V7

[96] Wong W. DREAM tools for contributors. doi:10.26180/14685651.V7

[97] Wong W, Andrade KC, Andrillon T, et al. DREAM: a Dream EEG and Mentation database. doi:10.31234/osf.io/69e43 PMC12350935 40804039

[98] Wong W. How to package Data for DREAM. doi:10.26180/13301498.V11

[99] Wong W. How to upload data to DREAM. doi:10.26180/13302218.V8

[100] Wong W. How to use DREAM report classifications. doi:10.26180/24032445.V1

[101] Kumral D, Palmieri J, Gais S, et al. 2023. doi:10.6084/M9.FIGSHARE.24794952.V1

[102] Elce V, Bergamo D, Avvenuti G, Bellesi M, Bernardi GLODE. 10.6084/M9.FIGSHARE.22147085.V1

[103] Motomura Y, Takeichi H, Ishii M, et al. MEG Kyushu. doi:10.6084/M9.FIGSHARE.27116590.V1

[104] Noreika V. Noreika_DATA1. doi:10.6084/M9.FIGSHARE.24058740.V1

[105] Noreika V, Windt J, Valli K, Revonsuo A. Lenggenhager B. noreika_motor_tDCS. doi:10.6084/M9.FIGSHARE.24058848.V1

[106] Lacaux C, Andrillon T, Arnulf I, Oudiette D. Oudiette_N1Data. doi:10.6084/M9.FIGSHARE.22210684.V1 PMC9677600 36415306

[107] Sikka P, Revonsuo A, Noreika V, Valli K. REM_Turku. doi:10.6084/M9.FIGSHARE.23274596.V2 PMC6561691 30988168

[108] Wong W, Andrillon T, Decat N, et al. The DREAM database. doi:10.26180/22133105.V5

[109] Siclari F, Baird B, Perogamvros L, Boly M, LaRocque J, Tononi G. Tononi serial awakenings. doi:10.26180/23306054.V2

[110] Konkoly K, Paller K, Mallett RTWC_USA. 10.6084/M9.FIGSHARE.22106123.V1

[111] Wamsley E, Zhang J, Collins MZ. Final. 2019. doi:10.6084/M9.FIGSHARE.22226692.V1

[112] R core team. R: a language and environment for statistical Computing. Available from:. https://www.R-project.org/. Accessed April 05, 2025.

[113] Posit team. RStudio: Integrated Development Environment for R. Posit Software, PBC, Boston, MA. 2024. Available from:. http://www.posit.co/. Accessed April 05, 2025.

[114] Benjamini Y, Hochberg Y. Controlling the false discovery rate: a practical and powerful approach to multiple testing. *J R Stat Soc Ser B Methodol*. 1995;57(1):289–300. doi:10.1111/j.2517-6161.1995.tb02031.x

[115] Lenth RV. emmeans: estimated MARGINAL MEANS, AKA LEAST-SQUARES MEans. Available from:. https://CRAN.R-project.org/package=emmeans. Accessed April 05, 2025.

[116] Ben-Shachar MS, Lüdecke D, Makowski D. effectsize: estimation of effect size indices and standardized parameters. *J Open Source Softw*. 2020;5(56):2815. doi:10.21105/joss.02815

[117] Venables WN, Ripley BD. *Modern Applied Statistics With S*. Fourth. Springer; 2002. https://www.stats.ox.ac.uk/pub/MASS4/.

[118] Kubinec R. Ordered beta regression: a parsimonious, well-fitting model for continuous data with lower and upper bounds. *Polit Anal*. 2023;31(4):519–536. doi:10.1017/pan.2022.20

[119] Brooks ME, Kristensen K, Benthem KJ, et al. glmmTMB balances speed and flexibility among packages for zero-inflated generalized linear mixed modeling. *R J*. 2017;9(2):378. doi:10.32614/RJ-2017-066

[120] Berry KJ, Johnston JE, Mielke PW Jr. Permutation methods. *WIREs Comput Stat*. 2011;3(6):527–542. doi:10.1002/wics.177

[121] Marzano C, Ferrara M, Mauro F, et al. Recalling and forgetting dreams: theta and alpha oscillations during sleep predict subsequent dream recall. *J Neurosci*. 2011;31(18):6674–6683. doi:10.1523/JNEUROSCI.0412-11.2011 21543596 PMC6632857

[122] Yu CK-C. Toward 100% dream retrieval by rapid-eye-movement sleep awakening: a high-density electroencephalographic study. *Dreaming*. 2014;24(1):1–17. doi:10.1037/a0035792

[123] St-Onge M, Lortie-Lussier M, Mercier P, Grenier J, De Koninck J. Emotions in the diary and REM dreams of young and late adulthood women and their relation to life satisfaction. *Dreaming*. 2005;15(2):116–128. doi:10.1037/1053-0797.15.2.116

[124] Sikka P, Valli K, Virta T, Revonsuo A. I know how you felt last night, or do I? Self- and external ratings of emotions in REM sleep dreams. *Conscious Cogn*. 2014;25:51–66. doi:10.1016/j.concog.2014.01.011 24565868

[125] Sterpenich V, Perogamvros L, Tononi G, Schwartz S. Fear in dreams and in wakefulness: evidence for day/night affective homeostasis. *Hum Brain Mapp*. 2020;41(3):840–850. doi:10.1002/hbm.24843 31663236 PMC7267911

[126] Daoust AM, Lusignan FA, Braun CMJ, Mottron L, Godbout R. Dream content analysis in persons with an autism spectrum disorder. *J Autism Dev Disord*. 2008;38(4):634–643. doi:10.1007/s10803-007-0431-z 17682931

[127] Schredl M, Hoffmann L, Sommer JU, Stuck BA. Olfactory stimulation during sleep can reactivate odor-associated images. *Chemosens Percept*. 2014;7(3):140–146. doi:10.1007/s12078-014-9173-4

[128] Palagini L, Gemignani A, Feinberg I, Guazzelli M, Campbell IG. Mental activity after early afternoon nap awakenings in healthy subjects. *Brain Res Bull*. 2004;63(5):361–368. doi:10.1016/j.brainresbull.2003.12.008 15245762

[129] Wamsley EJ, Hirota Y, Tucker MA, Smith MR, Antrobus JS. Circadian and ultradian influences on dreaming: a dual rhythm model. *Brain Res Bull*. 2007;71(4):347–354. doi:10.1016/j.brainresbull.2006.09.021 17208651

[130] Carr M, Nielsen T. Daydreams and nap dreams: content comparisons. *Conscious Cogn*. 2015;36:196–205. doi:10.1016/j.concog.2015.06.012 26164253

[131] Picard-Deland C, Pastor M, Solomonova E, Paquette T, Nielsen T. Flying dreams stimulated by an immersive virtual reality task. *Conscious Cogn*. 2020;83:102958. doi:10.1016/j.concog.2020.102958 32674062

[132] Wamsley EJ, Tucker M, Payne JD, Benavides JA, Stickgold R. Dreaming of a learning task is associated with enhanced sleep-dependent memory consolidation. *Curr Biol CB*. 2010;20(9):850–855. doi:10.1016/j.cub.2010.03.027 20417102 PMC2869395

[133] Lusignan FA, Zadra A, Dubuc MJ, Daoust AM, Mottard JP, Godbout R. Dream content in chronically-treated persons with schizophrenia. *Schizophr Res*. 2009;112(1–3):164–173. doi:10.1016/j.schres.2009.03.032 19409757

[134] Strauch I. REM dreaming in the transition from late childhood to adolescence: a longitudinal study. *Dreaming*. 2005;15(3):155–169. doi:10.1037/1053-0797.15.3.155

[135] Blagrove M, Edwards C, van Rijn E, et al. Insight from the consideration of REM dreams, non-REM dreams, and daydreams. *Psychol Conscious Theory Res Pract*. 2019;6(2):138–162. doi:10.1037/cns0000167

[136] Yu CK-C. We dream typical themes every single night. *Dreaming*. 2016;26(4):319–329. doi:10.1037/drm0000037

[137] Yu CK-C. We dream about typical themes in both REM and non-REM sleep. *Dreaming*. 2020;30(4):317–328. doi:10.1037/drm0000154

[138] Eichenlaub JB, van Rijn E, Gaskell MG, et al. Incorporation of recent waking-life experiences in dreams correlates with frontal theta activity in REM sleep. *Soc Cogn Affect Neurosci*. 2018;13(6):637–647. doi:10.1093/scan/nsy041 29868897 PMC6022568

[139] Vallat R, Nicolas A, Ruby P. Brain functional connectivity upon awakening from sleep predicts interindividual differences in dream recall frequency. *Sleep*. 2020;43(12):zsaa116. doi:10.1093/sleep/zsaa116 32597973

[140] Lara-Carrasco J, Nielsen TA, Solomonova E, Levrier K, Popova A. Overnight emotional adaptation to negative stimuli is altered by REM sleep deprivation and is correlated with intervening dream emotions. *J Sleep Res*. 2009;18(2):178–187. doi:10.1111/j.1365-2869.2008.00709.x 19645964

[141] Kusse C, Shaffii-Le Bourdiec A, Schrouff J, Matarazzo L, Maquet P. Experience-dependent induction of hypnagogic images during daytime naps: a combined behavioural and EEG study. *J Sleep Res*. 2012;21(1):10–20. doi:10.1111/j.1365-2869.2011.00939.x 21848802

[142] Chellappa SL, Münch M, Knoblauch V, Cajochen C. Age effects on spectral electroencephalogram activity prior to dream recall. *J Sleep Res*. 2012;21(3):247–256. doi:10.1111/j.1365-2869.2011.00947.x 21851439

[143] Sikka P, Revonsuo A, Sandman N, Tuominen J, Valli K. Dream emotions: a comparison of home dream reports with laboratory early and late REM dream reports. *J Sleep Res*. 2018;27(2):206–214. doi:10.1111/jsr.12555 28568911

[144] Wamsley EJ, Stickgold R. Dreaming of a learning task is associated with enhanced memory consolidation: replication in an overnight sleep study. *J Sleep Res*. 2019;28(1):e12749. doi:10.1111/jsr.12749 30091247 PMC6338510

[145] Schoch SF, Cordi MJ, Schredl M, Rasch B. The effect of dream report collection and dream incorporation on memory consolidation during sleep. *J Sleep Res*. 2019;28(1):e12754. doi:10.1111/jsr.12754 30091298 PMC6378621

[146] Zhang J, Wamsley EJ. EEG predictors of dreaming outside of REM sleep. *Psychophysiology*. 2019;56(7):e13368. doi:10.1111/psyp.13368 30912593 PMC6570568

[147] Wamsley EJ, Antrobus JS. Homeostatic and circadian influences on dreaming: NREM mentation during a short daytime nap. *Int J Dream Res*. 2008;1(2):27–33.

[148] Schredl M, Erlacher D. Is sleep-dependent memory consolidation of a visuo-motor task related to dream content? *Int J Dream Res*. 2010:, 74–79. doi:10.11588/ijodr.2010.1.486

[149] Nefjodov I, Winkler A, Erlacher D. Balancing in dreams: effects of playing games on the Wii balance board on dream content. *Int J Dream Res*. 2016:89–92. doi:10.11588/ijodr.2016.1.29034

[150] Klepel F, Schredl M. Correlation of task-related dream content with memory performance of a film task – a pilot study. *Int J Dream Res*. 2019:112–118. doi:10.11588/ijodr.2019.1.59320

[151] Pires J, Paiva TAS. Dream recall frequency and content in women. *Int J Dream Res*. 2021:266–271. doi:10.11588/ijodr.2021.2.80238

[152] Blagrove M, Fouquet NC, Henley-Einion JA, et al. Assessing the dream-lag effect for REM and NREM stage 2 dreams. *PLoS One*. 2011;6(10):e26708. doi:10.1371/journal.pone.0026708 22046336 PMC3202556

[153] Picard-Deland C, Nielsen T, Carr M. Dreaming of the sleep lab. *PLoS One*. 2021;16(10):e0257738. doi:10.1371/journal.pone.0257738 34614021 PMC8494361

[154] Wamsley EJ. Constructive episodic simulation in dreams. *PLoS One*. 2022;17(3):e0264574. doi:10.1371/journal.pone.0264574 35316266 PMC8939783

[155] Sikka P, Revonsuo A, Noreika V, Valli K. EEG frontal alpha asymmetry and dream affect: alpha oscillations over the right frontal cortex during REM sleep and presleep wakefulness predict anger in REM sleep dreams. *J Neurosci off J Soc Neurosci*. 2019;39(24):4775–4784. doi:10.1523/JNEUROSCI.2884-18.2019 PMC6561691 30988168

[156] Scarpelli S, D’Atri A, Bartolacci C, et al. Dream recall upon awakening from non-rapid eye movement sleep in older adults: electrophysiological pattern and qualitative features. *Brain Sci*. 2020;10(6):343. doi:10.3390/brainsci10060343 32503215 PMC7349242

[157] Grenier J, Cappeliez P, St-Onge M, et al. Temporal references in dreams and autobiographical memory. *Mem Cognit*. 2005;33(2):280–288. doi:10.3758/bf03195317 16028583

[158] Simon CW, Emmons W. EEG, consciousness, and sleep. *Science*. 1956;124:1066–1069. doi:10.1126/science.124.3231.1066 13380420

[159] Bonnet MH, Johnson LC, Webb WB. The reliability of arousal threshold during sleep. *Psychophysiology*. 1978;15(5):412–416. doi:10.1111/j.1469-8986.1978.tb01407.x 211536

[160] Abend G. What are neural correlates neural correlates of? *BioSocieties*. 2017;12(3):415–438. doi:10.1057/s41292-016-0019-y

[161] Vallat R, Lajnef T, Eichenlaub JB, et al. Increased evoked potentials to arousing auditory stimuli during sleep: implication for the understanding of dream recall. *Front Hum Neurosci*. 2017;11:11. doi:10.3389/fnhum.2017.00132 28377708 PMC5360011

[162] Leslie K, Skrzypek H, Paech MJ, Kurowski I, Whybrow T. Dreaming during anesthesia and anesthetic depth in elective surgery patients: a prospective cohort study. *Anesthesiology*. 2007;106(1):33–42. doi:10.1097/00000542-200701000-00010 17197843

[163] Cohen DB, MacNeilage PF. A test of the salience hypothesis of dream recall. *J Consult Clin Psychol*. 1974;42(5):699–703. doi:10.1037/h0036948 4372256

[164] Windt JM, Nielsen T, Thompson E. Does consciousness disappear in dreamless sleep? *Trends Cognit Sci*. 2016;20(12):871–882. doi:10.1016/j.tics.2016.09.006 27765517

[165] Alcaraz-Sánchez A, Demšar E, Campillo-Ferrer T, Torres-Platas SG. Nothingness is all there is: an exploration of objectless awareness during sleep. *Front Psychol*. 2022;13. doi:10.3389/fpsyg.2022.901031 PMC9226678 35756253

[166] Stumbrys T, Erlacher D. Lucid dreaming during NREM sleep: two case reports. *Int J Dream Res*. 2012;5(2):151–155.

[167] Alcaraz-Sanchez A. Awareness in the void: a micro-phenomenological exploration of conscious dreamless sleep. *Phenomenol Cogn Sci*. 2023;22(4):867–905. doi:10.1007/s11097-021-09743-0

[168] Koulack D, Goodenough DR. Dream recall and dream recall failure: an arousal-retrieval model. *Psychol Bull*. 1976;83(5):975–984. doi:10.1037/0033-2909.83.5.975

[169] Conduit R, Crewther SG, Coleman G. Shedding old assumptions and consolidating what we know: toward an attention-based model of dreaming. *Behav Brain Sci*. 2000;23(6):924–928. doi:10.1017/S0140525X00354024

[170] Conduit R, Crewther SG, Coleman G. Spontaneous eyelid movements (ELMS) during sleep are related to dream recall on awakening. *J Sleep Res*. 2004;13(2):137–144. doi:10.1111/j.1365-2869.2004.00397.x 15175093

[171] Koukkou M, Lehmann D. Dreaming: the functional state-shift hypothesis. A neuropsychophysiological model. *Br J Psychiatry J Ment Sci*. 1983;142:221–231. doi:10.1192/bjp.142.3.221 6860875

[172] Cohen DB, Wolfe G. Dream recall and repression: evidence for an alternative hypothesis. *J Consult Clin Psychol*. 1973;41(3):349–355. doi:10.1037/h0035333 4803268

[173] Schredl M. Dream recall researching dreams. *The Fundamentals*. 2018;11–34. doi:10.1007/978-3-319-95453-0_2

[174] Ackermann S, Hartmann F, Papassotiropoulos A, de Quervain DJF, Rasch B, de Quervain DJ-F. Associations between basal cortisol levels and memory retrieval in healthy young individuals. *J Cogn Neurosci*. 2013;25(11):1896–1907. doi:10.1162/jocn_a_00440 23806175

[175] Balkin TJ, Braun AR, Wesensten NJ, et al. The process of awakening: a PET study of regional brain activity patterns mediating the re‐establishment of alertness and consciousness. *Brain*. 2002;125(10):2308–2319. doi:10.1093/brain/awf228 12244087

[176] McNamara P, Auerbach S, Johnson P, Harris E, Doros G. Impact of REM sleep on distortions of self-concept, mood and memory in depressed/anxious participants. *J Affect Disord*. 2010;122(3):198–207. doi:10.1016/j.jad.2009.06.030 19631989 PMC2847051

[177] Goodenough DR, Lewis HB, Shapiro A, Jaret L, Sleser I. Dream reporting following abrupt and gradual awakenings from different types of sleep. *J Pers Soc Psychol*. 1965;2(2):170–179. doi:10.1037/h0022424 14316977

[178] Radulovic J, Lee R, Ortony A, Gonzálvez C, García-Fernández JM. State-dependent memory: neurobiological advances and prospects for translation to dissociative amnesia. *Front Behav Neurosci*. 2018;12:12. doi:10.3389/fnbeh.2018.00259 30429781 PMC6220081

[179] Raven F, Aton SJ. The engram’s dark horse: how interneurons regulate state-dependent memory processing and plasticity. *Front Neural Circuits*. 2021;15. doi:10.3389/fncir.2021.750541 PMC8473837 34588960

[180] Hobson JA, McCarley RW. The brain as a dream state generator: an activation-synthesis hypothesis of the dream process. *Am J Psychiatry*. 1977;134(12):1335–1348. doi:10.1176/ajp.134.12.1335 21570

[181] Wick AZ, Combertaldi SL, Rasch B. The first-night effect of sleep occurs over nonconsecutive nights in unfamiliar and familiar environments. *Sleep*. 2024;47(10):zsae179. doi:10.1093/sleep/zsae179 39126649 PMC11467056

[182] Demšar E, Windt J. Studying dream experience through dream reports: points of contact between dream research and first-person methods in consciousness science. doi:10.31231/osf.io/gxcym

[183] Thompson E. Dreamless sleep, the embodied mind, and consciousness. In: Metzinger T, Windt JM, editors. *Open MIND. Open MIND*. Frankfurt am Main: MIND Group; 2014. doi:10.15502/9783958570351.

[184] Bitbol M, Petitmengin C. Neurophenomenology and the Micro-phenomenological Interview. In: *The Blackwell Companion to Consciousness*. John Wiley & Sons, Ltd; 2017:726–739. doi:10.1002/9781119132363.ch51

[185] Nemeth G. The route to recall a dream: theoretical considerations and methodological implications. *Psychol Res*. 2023;87(4):964–987. doi:10.1007/s00426-022-01722-7 35960337

[186] Schredl M, Frauscher S, Shendi A. Dream recall and visual memory. *Percept Mot Skills*. 1995;81(1):256–258. doi:10.2466/pms.1995.81.1.256 8532466

[187] Nielsen T. Variations in dream recall frequency and dream theme diversity by age and sex. *Front Neurol*. 2012;3:106. doi:10.3389/fneur.2012.00106 22783222 PMC3389337

[188] Halliday G. Effect of encouragement on dream recall. *Dreaming*. 1992;2(1):39–44. doi:10.1037/h0094346

[189] Kunzendorf RG, Hartmann E, Cohen R, Cutler J. Bizarreness of the dreams and daydreams reported by individuals with thin and thick boundaries. *Dreaming*. 1997;7(4):265–271. doi:10.1037/h0094482

[190] Cavallero C, Cicogna P, Natale V, et al. Slow wave sleep dreaming. *Sleep J Sleep Res Sleep Med*. 1992;15(6):562–566. doi:10.1093/sleep/15.6.562 1475572

[191] Gardette J, Delhaye E, Bastin C. The multiple dimensions of familiarity: from representations to phenomenology. *WIREs Cogn Sci*. 2025;16(1):e1698. doi:10.1002/wcs.1698 39506460

[192] Mutz J, Javadi AH. Exploring the neural correlates of dream phenomenology and altered states of consciousness during sleep. *Neurosci Conscious*. 2017;2017(1):nix009. doi:10.1093/nc/nix009 30042842 PMC6007136

[193] Murdoch WJ, Singh C, Kumbier K, Abbasi-Asl R, Yu B. Definitions, methods, and applications in interpretable machine learning. *Proc Natl Acad Sci*. 2019;116(44):22071–22080. doi:10.1073/pnas.1900654116 31619572 PMC6825274

[194] Molnar C, Casalicchio G, Bischl B, et al. Interpretable machine learning – a brief history, state-of-the-art and challenges. In: Koprinska I, Kamp M, Appice A, editors. *ECML PKDD 2020 Workshops*. Springer International Publishing; 2020:417–431. doi:10.1007/978-3-030-65965-3_28

[195] Stucky B, Scholz M, Lakämper S, Keller K, Kreamer T, Landolt HP. Towards multimodal biomarker discovery of sleep deprivation with interpretable machine learning. *J Sleep Res*. 2024;33(S1):P581. doi:10.1111/jsr.14291

[196] Kuhn RL. A landscape of consciousness: toward a taxonomy of explanations and implications. *Prog Biophys mol Biol*. 2024;190:28–169. doi:10.1016/j.pbiomolbio.2023.12.003 38281544

[197] Masi M. An evidence-based critical review of the mind-brain identity theory. *Front Psychol*. 2023;14. doi:10.3389/fpsyg.2023.1150605 PMC10641890 37965649

[198] Konkoly KR, Appel K, Chabani E, et al. Real-time dialogue between experimenters and dreamers during REM sleep. *Curr Biol CB*. 2021;31(7):1417–1427.e6. doi:10.1016/j.cub.2021.01.026 33607035 PMC8162929

[199] Türker B, Musat EM, Chabani E, et al. Behavioral and brain responses to verbal stimuli reveal transient periods of cognitive integration of the external world during sleep. *Nat Neurosci*. 2023;26(11):1981–1993. doi:10.1038/s41593-023-01449-7 37828228 PMC10620087

[200] Cornelissen M. In defence of rigorous subjectivity. *Transpers Psychol Rev*. 2007;11(1):8–18. doi:10.53841/bpstran.2007.11.1.8

[201] Cornelissen M. The need for the Indian tradition. *Psychol Stud*. 2003;48(3):38–52.

[202] Sri Aurobindo. The Science of Consciousness. *Essays Divine and Human*. Vol. 12.1993rd. The Complete Works of Sri Aurobindo. Sri Aurobindo Ashram Publication Department;1997. 323

